# Delineating SARS-CoV-2 spike protein and antibodies interaction interfaces via siamese neural networks: A geometric and image-based analysis

**DOI:** 10.1371/journal.pone.0335270

**Published:** 2025-11-04

**Authors:** Gemma Loreti, Paola Vottero, Elena Carlotta Olivetti, Enrico Vezzetti, Jack Tuszynski, Federica Marcolin, Maral Aminpour

**Affiliations:** 1 Department of Management and Production Engineering, Politecnico di Torino, Corso Duca degli Abruzzi, Turin, Italy; 2 Department of Biomedical Engineering, University of Alberta, Edmonton, Alberta, Canada; 3 Department of Mechanical and Aerospace Engineering, Politecnico di Torino, Corso Duca degli Abruzzi, Turin, Italy; National University of Singapore, SINGAPORE

## Abstract

The analysis of molecular interactions between antigens and antibodies is crucial for understanding the immunological mechanisms underlying the immune response and for developing effective therapies against various diseases. In this context, the ability to distinguish between protein interfaces that form stable and unstable complexes is a key step in the design of therapeutic antibodies and vaccines. In recent years, deep learning models have provided advanced tools for biomedical research. This work introduces a novel approach to analyzing antibody-antigen interactions, and in particular SARS-CoV-2 spike protein-targeting antibodies, using a Siamese Neural Network specifically designed to integrate depth maps with geometric descriptors of molecular surfaces. By combining these representations, the model captures geometrical shape complementarity to differentiate between stable and unstable protein complexes. The network was trained using image-based representations of antigens and antibodies interfaces enriched with geometric descriptors, using data that include binders and non-binders of the SARS-CoV-2 spike protein receptor-binding domain. The deep learning network operates by comparing feature vectors representing these molecular surfaces; pairs with closer vectors in feature space are associated with stable interactions, while those with more distant vectors suggest instability. Extensive testing with different configurations achieved an accuracy of 90%, demonstrating the robustness of this approach to predict interactions. This innovative integration of artificial intelligence, depth maps and geometric descriptors offers promising applications for designing novel antibodies and vaccines.

## Introduction

Protein-protein interactions (PPIs) are fundamental to understanding vital cellular processes, such as enzyme regulation and signaling. The ability to predict which proteins form complexes with each other is crucial for biomedical research, as PPIs are directly involved in many diseases and are strategic targets for drug development.

Antibodies, or immunoglobulins, are proteins produced by the immune system to identify and neutralize pathogens such as viruses and bacteria. Each antibody consists of two light chains and two heavy chains, forming two main functional regions: Fab (fragment antigen-binding) and FC (fragment crystallizable). The Fab region contains the antigen-binding site, responsible for the specific recognition of antigenic molecules. When an antibody binds to an antigen ant, it can directly neutralize it or mark the pathogen for elimination by other components of the immune system [1,2]. In the case of a virus like SARS-CoV-2, responsible for the COVID-19 pandemic, the virus uses its spike protein (S) to bind to receptors on host cells, triggering infection. The spike protein is composed of two main subunits, S1 and S2, with the receptor-binding domain (RBD) located within the S1 subunit. Antibodies can bind to the RBD of the spike protein, thus preventing the virus from attaching to the ACE2 receptor on human cells, blocking viral entry and thereby neutralizing the infection [3].

**Fig 1:**
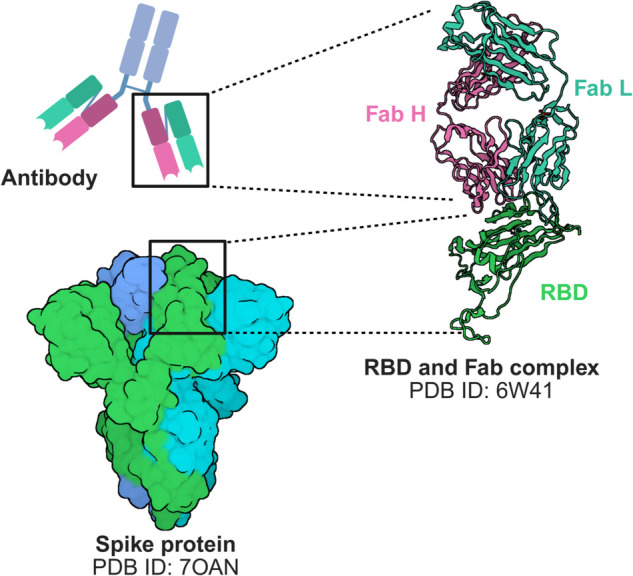
Structural context for RBD–antibody binding. Left: SARS-CoV-2 spike protein (PDB 7OAN), shown as a cartoon, and schematic of an antibody structure. Right: the spike receptor-binding domain (RBD) bound by a representative Fab (PDB 6W41); heavy (H) and light (L) chains are labeled. The cartoon views emphasize the antigen-binding interface on the RBD and the Fab paratope formed by the CDR loops, providing structural context for our 2D inputs (grayscale surface images) and the per-pixel descriptor maps used throughout the study. Created with BioRender.com.

The area or interface between the two proteins (an example is shown in Fig 1 for a spike RBD-antibody pair) is the contact point where their molecular surfaces meet and interact, forming a complex. These interaction areas consist of amino acid residues that can establish various types of bonds (such as hydrogen bonds, hydrophobic interactions, ionic bonds, or Van der Waals forces), therefore determining the strength and stability of the complex [4]. Understanding the chemical structure of interfaces involves examining the types of interactions and the chemical properties of the involved protein surfaces, such as the distribution of electric charges, polarity, and the presence of reactive residues. In parallel, the geometric aspect concerns the three-dimensional shape of the interface, its complementarity and its accessibility. Indeed, the two surfaces must fit together optimally for the interaction to be effective; if the geometry is not favorable, i.e., the two surfaces are not shape-complementary, the interaction will be weak or non-existent [5].

Over the years, several approaches, both experimental and computational, have been developed to study and characterize these types of interactions. In the early 1980s, Kuntz et al. [6] pioneered a geometry-based approach to analyze biomolecular interactions, using geometric alignment of rigid-body representations for proteins and ligands (approximated by spheres) to identify compatible binding sites and model protein-ligand interactions based on spatial and molecular compatibility. Jones and Thornton [7] used a combined scoring system focused on interface residues to predict protein-protein interaction sites. Later, Lee et al. [8] refined this by classifying the binding sites according to the geomorphic features of the Earth (cave, crater, canyon, plain, and valley).

Computational approaches have gained ground thanks to the increase in computing power and the availability of large datasets. Molecular docking, sequence analysis, and protein structure modeling have become key tools for predicting and analyzing interactions. Furthermore, the application of machine learning methods has opened new frontiers in PPI prediction, allowing for more accurate and sophisticated models. The emergence of geometric deep learning, which, unlike traditional deep learning (focused on structured data like grids or sequences), can handle complex non-Euclidean data structures, including graphs and surfaces, was a significant development. However, many of these methods primarily rely on either sequence data or structural abstractions, often neglecting detailed geometric representations of binding interfaces. A notable mention in PPI prediction is the application of Siamese neural networks. Introduced in the 1990s for signature verification [9], Siamese architectures have become integral to tasks like face recognition, image retrieval, and object tracking, particularly after their successful application in one-shot learning by Koch et al. [10]. In PPI prediction, Siamese networks have been adapted to model protein sequence relationships and structural information to capture subtle but essential differences between interacting protein pairs.

Several models have been proposed using protein sequences as inputs, or features engineered thereof. Chen et al. implemented a Siamese network model using pairs of protein sequences to capture mutation effects on binding affinity. This model outperformed traditional sequence-based models such as support vector machines (SVM), k-nearest neighbors (kNN), and random forest by providing binary PPI predictions along with multiclass interaction type classification and binding affinity estimation [11]. Yang et al. further improved Siamese networks for PPI prediction by integrating a transfer learning module, enhancing performance in human-virus PPI predictions, including SARS-CoV-2, by combining pre-acquired evolutionary sequence features with a Siamese CNN and a multilayer perceptron (MLP) [12]. Mahapatra et al. developed a hybrid classifier combining a Siamese network with a light gradient boosting machine (LGBM) algorithm. Their model leverages descriptors like pseudo-amino acid composition (PseAAC) and conjoint triad (CT) to transform variable-length protein sequences into fixed-length numerical arrays. This approach enabled accurate PPI network reproduction, as well as high-performance predictions across various metrics [13]. Wu et al. introduced a Siamese Pyramidal network inspired by feature pyramid networks (FPNs) [14], incorporating an attention module to account for protein folding and self-binding, optimizing prediction accuracy by analyzing primary sequences and spatial conformations [15]. Chen et al.’s 2022 model leveraged natural language processing-inspired encoding to convert amino acids into vectors and predict binary PPI classifications [16]. This model applied a dissimilarity threshold to identify negative samples, ensuring non-homologous protein comparisons by removing sequences with more than 40% similarity, as introduced by Eid et al. [17]. Madan et al. expanded on these methods by integrating Elnaggar et al.’s ProtBERT [18] deep sequence embedding model into a Siamese network architecture. Their model provides binary classification and multiclass predictions for interaction types, with binding affinity scores and explainability features using integrated gradients to track the influence of individual amino acids on the model predictions [19].

A notable limitation of these models is the lack of local spatial and structural information; as mentioned above, the geometrical compatibility between protein pairs is an important driving force for binding.

Geometric deep learning approaches for PPI prediction have emerged that take into account the structural information of proteins. Gainza et al. developed the MaSIF framework, applying geometric deep learning to molecular surfaces and hypothesizing that proteins with similar interaction functions may share ’fingerprint’ patterns on their surfaces, regardless of their evolutionary background. MaSIF addresses three tasks: prediction of protein-ligand binding sites, identification of PPI sites, and scanning of protein surfaces to predict PPI complexes [20,21]. Later, the authors introduced a framework for real-time protein surface learning with efficient geometric convolutional layers, followed by a de novo protein interaction design model that generates molecular surface descriptors to guide interaction predictions [22]. Dai and Bailey-Kellogg introduced PInet, a neural network model based on point clouds encoding structural information of protein pairs. This model captures both geometric and physicochemical complementarity between protein surfaces, providing information on which regions mediate interactions [23]. Das and Chakrabarti also contributed with a machine learning classifier, built on support vector machines, aimed at differentiating native from non-native complexes [24]. DockNet, a Siamese graph neural network model, addresses limitations in traditional docking methods, which often struggle with protein flexibility. DockNet predicts contact residues at the protein interfaces by using graph representations where nodes represent amino acid residues, and edges capture chemical or spatial relationships, providing residue-level predictions that incorporate flexibility [25]. Recent developments include the PeSTo framework, a parameter-free approach to geometric deep learning for the prediction of binding interfaces, which avoids the reliance on predefined thresholds or heuristics by leveraging geometric insights to improve prediction accuracy [26]. The EGGNet framework extends geometric learning to various molecule types interacting with protein targets, accommodating small molecules, synthetic peptides, and natural proteins, thereby expanding the applicability of the model to diverse molecular interactions [27].

In this work, we propose an AI-based approach using images within a Siamese neural network, specifically designed to predict SARS-CoV-2 spike-targeting antibodies. Understanding these interactions is crucial for COVID-19 therapeutic and vaccine development, and our model is tailored for this purpose. To achieve this, we curated a dataset containing both spike protein binders and non-binders, ensuring that the model distinguishes between interacting and non-interacting pairs. Unlike existing approaches that focus solely on sequence or structural abstraction, our method uniquely combines precise spatial features of binding interfaces. Our model exploits a representation of protein interfaces in the form of depth maps and descriptors that capture the crucial geometric features of the protein surface. The network, a deep learning architecture specifically designed to learn similarity relations between input pairs, is optimized to discriminate between proteins that form a complex and those that do not. This dual-layered approach improves predictive power and reduces the risk of bias. The core of the network training is the use of contrastive loss, an optimization technique specific to Siamese networks, which encourages the network to learn spatial representations where protein pairs that form complexes are close to each other in the feature space, while those that do not are far apart. Our high test accuracy supports that the model effectively captures meaningful patterns relevant to antibody-spike interactions rather than memorizing known structures. While broader applications may be explored in future research, this study deliberately focuses on COVID-19 spike-targeting antibodies, with the chosen dataset being specifically designed for this scope. The proposed framework represents a valuable step forward in leveraging deep learning for targeted biomedical applications, offering potential insights into SARS-CoV-2 therapeutic strategies.

## Materials and methods

### Data retrieval

The three-dimensional structures of antibody-antigen complexes were obtained from the Structural Antibody Database (SAbDab) [28], which compiles all antibody structures available in the Protein Data Bank (PDB) [29], and the Coronavirus Antibody Database CoV-AbDab [30] from the Oxford Protein Informatics Group (OPIG). A total of 637 PDB files representing antibodies targeting the spike protein’s receptor-binding domain (RBD) were obtained from SAbDab by filtering for entries also listed in CoV-AbDab with the RBD as the binding partner. Additionally, 711 structures of antibodies identified as non-spike binders were collected by excluding entries containing spike protein in CoV-AbDab. Further filtering retained only the PDB files with complexes (structures where the Fab of the antibody is co-crystallized with the antigen) yielding 442 and 626 structures for spike binders and non-binders, respectively. The SAbDab’s summary file, which provides chain information for each structure, allowed us to separate complexes into two PDB files representing the Fab and antigen components. The Bio.PDB module from Biopython [31], a suite of Python tools for bioinformatics, facilitated this process.

### Surface representation

The TMSmesh v2.1 software [32] was used to generate triangular mesh representations of protein surfaces. This process began with converting PDB files to PQR format, which includes charge and radius information, using PDB2PQR [33]. TMSmesh produces a Gaussian surface, a smooth approximation of the protein’s electron density created by Gaussian functions around each atom [34,35]. Two key parameters, the decay rate *d* and the isovalue *c*, control the shape and volume of this surface. TMSmesh was tested against other popular meshing tools like MSMS, Molsurf, and NanoShaper, demonstrating robust handling of large biomolecules and producing watertight meshes. For specific approximations, *d* and *c* were tuned to match van der Waals surfaces (*d* = 2.0, *c* = 1.0), solvent-accessible surfaces (SAS), and solvent-excluded surfaces (SES) (*d* values between 0.4 and 0.6 and *c* values around 1.7). Version 2.1 integrates TMSmesh2.0’s trilinear surface approximation of the Gaussian functions for faster computation and a mesh quality enhancement routine [32,36]. Here, the parameter *e*, which controls the precision between the trilinear and Gaussian representations, was set at 0.99, consistent with the authors’ indication, while the decay rate *d* was set to 0.5 to approximate SES.

### Interface extraction

As the dataset consists of Fab-antigen complexes, interfacing patches could be extracted from the meshes representing the two proteins in the complex. A distance-based approach was employed to do so; given entities *A* and *B* in a complex *C*, the following steps were performed to identify the interface vertices within protein *B*:

- A three-dimensional tree was built using the scikit-learn package KDTree to represent protein *A*’s vertices;
- KDTree’s query functionality was used to compute pairwise distances between the vertices of *A* and *B* by using *B* as a query;
- The distances were filtered using *thr* = 2 Å as a threshold, so that *B*’s vertices less than *thr* from *A* were retained as part of the interface patch.

The resulting dataset comprises three-dimensional coordinates that form point clouds of antigen–antibody interfaces. Positive pairs were defined as co-crystallized RBD–Fab complexes (binders), whereas negative pairs were obtained by combining RBD structures with non-cognate Fabs (non-binders). For each pair, we extracted two interface point clouds: the RBD interface and the Fab interface.

### Image preparation

Interface point clouds were initially positioned arbitrarily in 3D. To obtain consistent orientations across complexes, each interface (RBD and Fab) was independently aligned via principal component analysis: centered coordinates were subjected to Singular Value Decomposition (SVD) to identify principal axes [37], and the point cloud was rotated to align its dominant directions with the Cartesian axes. This choice optimizes the *z*-to-*xy* projection, yielding a more descriptive 2D distribution while keeping antigen and Fab orientations consistent across samples, despite being processed independently.

Next, two types of representations were rendered for each protein interface using the nearest-neighbor interpolation method [38], which is effective in providing a continuous representation of surfaces while preserving local structural details. The first representation, referred to as the *normal* interface, was generated by directly applying Nearest-Neighbor interpolation to the point cloud data. The second representation, called the *smooth* interface, was obtained by applying Nearest-Neighbor interpolation followed by a smoothing filter with a spherical kernel. The filter is developed and presented in [39]. This filter uses a spherical kernel with a radius of 0.6 nm to mimic molecular interactions with the studied protein, inspired by the spherical interaction model proposed by Kuntz et al. [6] and helps reduce noise and irregularities, creating a smoother surface. The network was tested using both the normal and smooth representations to determine which one provided a better understanding of the protein interface.

From each of the two interpolated versions of every protein, a depth map was calculated. Depth maps provide a two-dimensional representation of the original three-dimensional structures, capturing variations in depth to highlight the shape and topology of the molecular surface. This representation is particularly useful for identifying structural features such as pockets, cavities, or exposed regions of the protein. To ensure consistency, the depth map images were resized according to the average width and height among all images (470*x*345 pixels), and were processed as one-channel images (grayscale) with intensities normalized per-pair (using pair-shared min–max bounds).

#### Data augmentation.

The following data augmentation techniques were tested:

- Geometric flip: coordinates were transformed as z→−z before depth map creation, to obtain a mirrored patch across the *xy* plane;
- In-plane rotation: 180° rotation in the *xy* plane;
- Horizontal and vertical flips.

These transformations conserved the geometry (curvatures, concavities, convexities) while expanding the image space by a factor of 4. Augmentations were used only for training, while leaving the validation and test sets unaugmented. Augmentations were paired-consistent (the same transform was applied to Fab and RBD images in each pair), and intensity scaling used pair-shared min–max bounds. An ablation experiment was performed to assess the impact of data augmentation on the performance of the model.

### Geometrical descriptors

Differential geometry descriptors have been mapped on the proteins’ depth maps to retrieve their shape information. Here, we use a set of geometric descriptors that have been successfully applied in previous research regarding 3D human face analysis [40] and protein shape complementarity [41,42]. Specifically, eight descriptors were selected to characterize the geometry and topology of the protein surfaces (described in [40]): mean curvature (denoted as *H*), principal curvatures (*k*_1_ and *k*_2_), Gaussian curvature (*K*), shape index (*S*), curvedness (*C*), the sine of the third coefficient of the second fundamental form (*sing*) and a descriptor that highlights symmetry properties inspired by the second coefficient of the first fundamental form called *F*_*den*2_. These descriptors depend on the surface derivatives, enabling them to effectively illustrate alterations in surface curvature, surface depressions and peaks, and symmetry aspects of the surface itself.

The six primary descriptors arise from the first and second fundamental forms:

$$ds^{2}=E\;du^{2}+2F\;dudv+G\;dv^{2},$$ (1)

$$ds^{2}=e\;du^{2}+2f\;dudv+g\;dv^{2},$$ (2)

where *E*,*F*,*G*,*e*,*f*,*g* are the coefficients defined by:

$$E=1+h_{x}^{2},$$ (3)

$$F=h_{x}h_{y},$$ (4)

$$G=1+h_{y}^{2},$$ (5)

$$e=\frac{h_{xx}}{\sqrt{1+h_{x}^{2}+h_{y}^{2}}},$$ (6)

$$f=\frac{h_{xy}}{\sqrt{1+h_{x}^{2}+h_{y}^{2}}},$$ (7)

$$g=\frac{h_{yy}}{\sqrt{1+h_{x}^{2}+h_{y}^{2}}},$$ (8)

where *h* denotes a differentiable function representing the three-dimensional surface, and *h*_*x*,*y*,*xx*,*yy*,*xy*_ are the surface derivatives.

Curvatures are used to measure how a surface bends. The principal curvatures *k*_1_ and *k*_2_ are the roots of the following quadratic equation:

$$x^{2}-2Hx+K=0.$$ (9)

Thus, *k*_1_ and *k*_2_ are given by:

$$k_{1}=H+\sqrt{H^{2}-K},$$ (10)

$$k_{2}=H-\sqrt{H^{2}-K},$$ (11)

where *K* and *H* represent the Gaussian and mean curvatures, respectively, defined as follows:

$$K=\frac{eg-f^{\;2}}{EG-F^{2}}=k_{1}k_{2},$$ (12)

$$H=\frac{eG-2fF+gE}{2(EG-F^{2})}=\frac{k_{1}+k_{2}}{2}.$$ (13)

The shape index *S*, which characterizes the surface shape, and the curvedness index *C*, providing a measure of the curvature, were defined by Koenderink and van Doorn [43] as:

$$S=-\frac{2}{\pi }\arctan\left(\frac{k_{1}+k_{2}}{k_{1}-k_{2}}\right),\;S\in [-1,1],\;k_{1}\leq k_{2},$$ (14)

$$C=\sqrt{\frac{k_{1}^{2}+k_{2}^{2}}{2}}.$$ (15)

The descriptor *F*_*den*2_ is a derived descriptor that was formulated as [40]:

$$F_{den2}=\frac{F}{1+h_{x}^{2}+h_{y}^{2}}.$$ (16)

The descriptors have been evaluated point by point on the depth maps. In some cases (*k*_1_, *k*_2_, *g*, *H*, *S*), their mean- or median-filtered versions have been employed in place of the original descriptors for smoothing purposes, as described in [40].

### Siamese neural network and training objective

Siamese neural networks learn to compare two inputs by mapping them into a shared feature space with weight-tied subnetworks and then evaluating a similarity measure [44]. In our setting, each input image *x* (RBD or Fab) may contain an image channel and, optionally, descriptor channels. We therefore employ two parallel modality towers per branch: an image tower and a descriptor tower. The image tower consists of three convolutional blocks:

-
Conv2d(k=10,stride2)→BatchNorm→LeakyReLU(0.01)→MaxPool(3×3,stride2)

-
Conv2d(k=5,stride2)→BatchNorm→LeakyReLU→MaxPool(2×2)

-
Conv2d(k=3,stride1)→BatchNorm→LeakyReLU→MaxPool(2×2)

**Fig 2:**
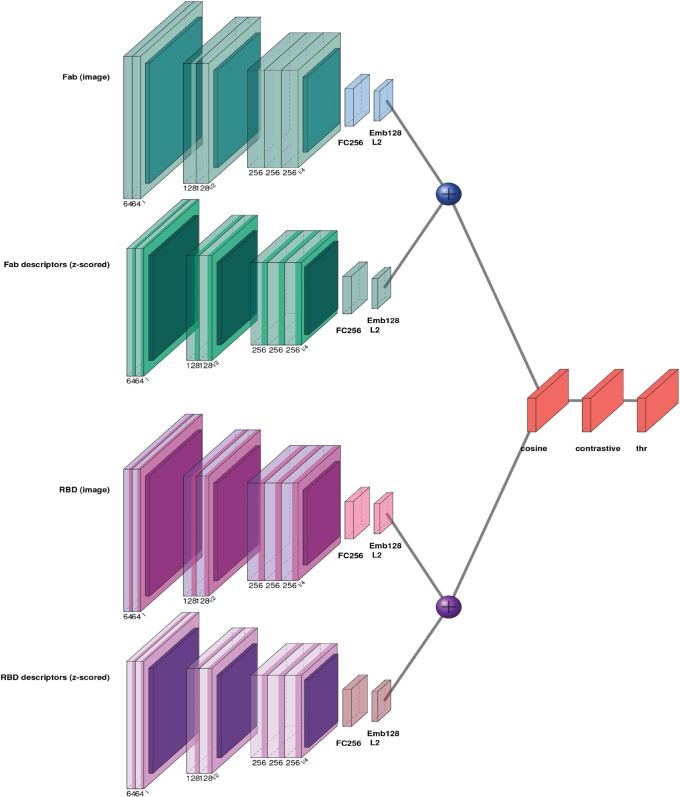
Overview of the Siamese two-tower architecture used. For each item (Fab and RBD), the raw image (top/third rows) and the z-scored descriptor stack (second/fourth rows) are encoded by CNNs. The image and descriptor embeddings are fused (⊕, implemented as a small MLP) to produce a single representation. The two fused embeddings are compared with cosine similarity and converted to a distance metric *d* = 1−*cosine*; training minimizes a contrastive loss with margin *m* = 1.0, and the operating threshold *thr* is chosen on the validation ROC. Channel counts are annotated beneath blocks; aqua tones denote Fab branches and purple tones denote RBD branches.

A small fully connected head follows: Linear→256→BatchNorm→LeakyReLU→Dropout(p=0.1)→Linear→d, with the output *L*_2_-normalized to yield $z^{img}\in {\mathbb{R}}^{d}$ (*d* = 128). The descriptor tower has the same layout except for the first convolution (k=7,stride2), producing *z^desc^*. LeakyReLU is used throughout to avoid zero-gradient saturation and improve early optimization. When only one modality is present, we use its embedding directly. When both are present, we apply concat fusion: $\left[z^{img};z^{desc}\right]$ passes through a small fusion multi-layer perceptron (MLP, Linear2d→2d→BatchNorm→LeakyReLU(0.01)→Dropout(0.1)→Linear2d→d) and the result is *L*_2_-normalized to obtain the fused embedding *z*. A paired forward pass encodes $\left(x_{1},x_{2}\right)$ into $\left(z_{1},z_{2}\right)$ with shared weights across branches. Similarity is computed via cosine similarity:

$$s=\frac{\langle z_{1},z_{2}\rangle }{\|z_{1}{\|}_{2}\|z_{2}{\|}_{2}}$$ (17)

with cosine distance d=1−s∈[0,2]. Training uses a cosine contrastive loss, defined as:

$$ℒ=yd^{2}+(1-y)[max(0,m-d){]}^{2}$$ (18)

where y∈{0,1} indicates binders (1) versus non-binders (0), and *m* is a margin (set to 1.0). The objective pulls embeddings of binding pairs together (d→0) while pushing non-binding pairs at least *m* apart. A schematic representation of the architecture is shown in Fig 2.

Training was performed with the Adam optimizer (learning rate 1*e*^−4^) and Xavier initialization. Unless otherwise noted, inputs are depth maps; two- or more-channel variants concatenate one or more geometric descriptors with the depth map. For analysis and calibration, we also expose per-modality embeddings to compute image-only and descriptor-only distances; at validation, when both modalities are available, we apply late fusion by linearly blending these distances with a weight chosen to maximize ROC–AUC. PyTorch was used for implementation and training [45–47]. A class-balanced sampler ensured equal representation of binders and non-binders in each batch. For evaluation, cosine distances were converted into binary classifications using thresholds selected on the validation set, as described below.

### Dataset splitting and evaluation

#### Homology-aware data splits.

As a pairwise learning framework trained on images was employed, our approach does not rely on sequence information; instead, it leverages geometric and spatial features extracted from depth maps and geometric descriptors of antigen-antibody interfaces. As a result, sequence homology does not directly influence the learning process, since the model does not receive sequence data as input. Nonetheless, we adopted a homology-aware split to reduce potential information leakage arising from highly similar antibodies across folds. We implemented this at the CDR-H3 level, as H3 is the most diverse and paratope-defining region. Heavy-chain CDRs were extracted from FASTA sequences using ANARCI [48] with IMGT numbering, with a robust fallback to index-based slicing when region labels were unavailable. For homology grouping, we retained CDR-H3 sequences and wrote per-antibody H3 FASTAs. We clustered H3 sequences with CD-HIT [49] at 90% identity and 80% coverage, yielding H3 clusters that served as the group units for splitting. This enforces that antibodies sharing ≥90% H3 identity are assigned to the same split. To reduce label imbalance before splitting, we undersampled the global majority label (non-binding antibodies) at the H3-group level while preserving H3 diversity. Concretely, we selected one representative H3 per majority group, meta-clustered these representatives with CD-HIT at 95% identity, then greedily retained at most one (and, if needed, additional) group per meta-cluster until the target majority proportion of 50% was reached. All minority-label groups were kept. We performed a stratified split of H3 clusters into 70%/20%/10% train/validation/test sets, optimizing (i) total weight per split, (ii) per-split label proportions within a tolerance (default ±0.10) relative to the global label distribution, and (iii) a minimum number of groups in validation and test (defaults 30/30). The procedure iterates many random orders of groups and selects the assignment minimizing a size-and-label deviation objective, subject to the constraints. After the split, we quantified potential leakage by computing, for each validation/test H3, the maximum identity to any training H3 using a simple global aligner (match 1, mismatch 0, gap 0). The median of these nearest-neighbor identities was ∼60% for both validation and test, indicating limited homology to the training examples. Grouping by CDR-H3 identity captures the principal source of paratope similarity, thereby limiting near-duplicate antibodies from straddling splits. The stratified, group-aware optimization preserves overall label balance while enforcing this homology constraint.

#### Positive/negative pairs.

Positive pairs were defined as co-crystallized RBD–Fab complexes (binders, label 1), whereas negative pairs were obtained by pairing RBD structures with non-cognate Fabs (non-binders, label 0). For each pair, we extracted two interface representations (RBD interface and Fab interface), rendered as depth maps and, where applicable, concatenated with one or more geometric descriptors.

#### Evaluation.

Model selection and calibration were performed on the validation set. Cosine distances were converted to binary predictions using Youden’s 𝒥 statistic, defined as:

𝒥=TPR−FPR (19)

where TPR is the true positive rate (sensitivity) and FPR is the false positive rate (1 - specificity). The threshold maximizing 𝒥 on the validation set was selected, then fixed for the test set to ensure unbiased evaluation. We report mean ± standard deviation of accuracy, precision, recall, F1, and ROC–AUC across five independent seeds.

**Fig 3:**
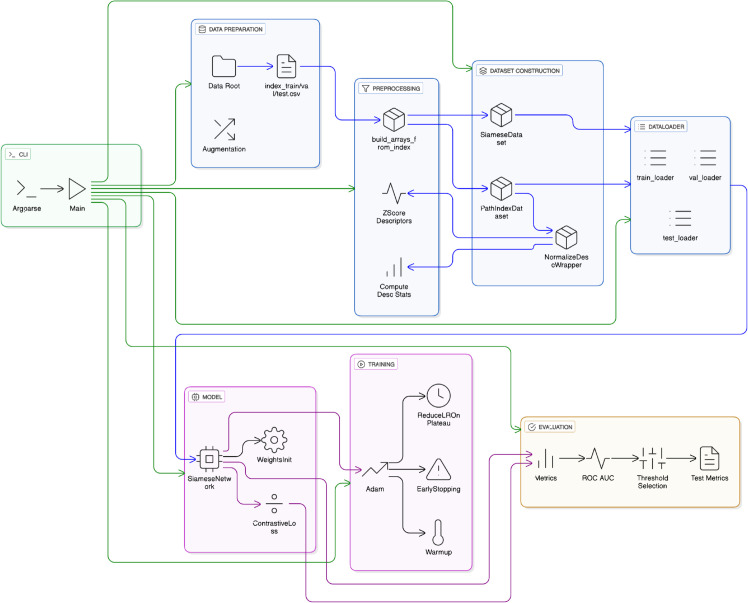
End-to-end pipeline for training and evaluation. Schematic overview of the modular framework implemented in this work. Each block corresponds to a distinct stage of the workflow, with the internal labels reflecting the actual classes and functions implemented in the code. From left to right: (i) CLI and Data Preparation: command-line arguments specify dataset root and index files (index_train/val/test.csv), with optional interface augmentations. (ii) Preprocessing: geometric descriptors are z-scored, either eagerly or on-the-fly, to standardize input channels. (iii) Dataset Construction: inputs are wrapped into dataset classes that enable flexible handling of preloaded and lazy modes. (iv) Dataloaders: balanced train/validation/test loaders are created to enforce class balance across epochs. (v) Model: a Siamese neural network encodes Fab and RBD interfaces into a shared embedding space, combining image and descriptor representations via fusion. Initialization and training rely on weights_init and ContrastiveLoss. (vi) Training: optimization is performed with Adam, with warmup, ReduceLROnPlateau scheduling, and early stopping on validation AUC. (vii) Evaluation: cosine distances are converted to binary predictions using Youden’s 𝒥 statistic, with final metrics (accuracy, precision, recall, F1, ROC–AUC) reported on the test set.

An overview of the adopted pipeline is provided in Fig 3.

### Ablation experiments

To evaluate the contribution of individual components of our pipeline, we conducted a series of ablation experiments. All ablations were trained under identical conditions (five seeds, balanced batches, early stopping on validation AUC) to ensure comparability. The following parameters were ablated:

- Loss formulation: the final cosine-based contrastive loss was compared to the standard Euclidean contrastive formulation. This tested whether cosine distance (aligned with our L2-normalized embeddings) was necessary for stable training and meaningful similarity learning.
- Fusion strategy: within the Siamese architecture, we ablated the fusion MLP that combines image and descriptor embeddings after concatenation, to determine whether it improved cross-modal integration beyond simple concatenation.
- Data augmentation: experiments were run with and without paired-consistent augmentations (rotations and flips).
- Surface preprocessing: normal and smooth interface representations were tested in the image-only input setting to assess the effect of smoothing.
- Input modality: we compared depth maps alone, depth maps combined with each descriptor individually, and depth maps with all descriptors jointly. Additionally, a greedy forward selection was implemented to identify a minimal optimal subset of descriptors that improved performance above the baseline. This clarified whether geometric descriptors provide complementary information beyond the shape-based depth representation.

These ablations collectively support the design rationale for the final model: a cosine-based contrastive Siamese network operating on depth + descriptor inputs with learned cross-modal fusion.

### Baseline comparison

As a comparative baseline, we implemented a sequence-only Siamese network. Antibody sequences were numbered using ANARCI (IMGT scheme), from which complementarity-determining regions (CDRs) were extracted. Sequences were embedded with the pretrained ESM-3 protein language model (frozen) [50], and embeddings were passed through a Siamese MLP trained with a contrastive objective. We evaluated three input variants: all six CDRs, heavy-chain CDR3 only, and full Fab sequences.

## Results and discussion

### Ablations results

#### Loss formulation.

We compared the standard Euclidean contrastive loss with the cosine-based variant. As shown in Table 1, the Euclidean formulation led to substantially lower accuracy and AUC, with higher variance across seeds. In contrast, the cosine-based loss consistently achieved more stable convergence and improved discrimination between binding and non-binding interfaces. This indicates that cosine distance is a better match for the normalized embedding space learned by our Siamese architecture.

**Table 1:** Ablation results comparing Euclidean and cosine contrastive loss functions. Metrics are reported on the test set as mean ± standard deviation across five seeds. Best values per metric are highlighted in bold.

Loss	Accuracy	Precision	Recall	F1 Score	AUC
Cosine	0.91±0.01	0.86±0.01	0.99±0.01	0.92±0.01	0.95±0.005
Euclidean	0.81±0.04	0.75±0.05	0.92±0.03	0.82±0.03	0.90±0.01

#### Fusion strategy.

We next evaluated the effect of feature fusion when combining depth maps with geometric descriptors. The tests were conducted using all descriptors as input, without data augmentation. The results of the ablation are reported in Table 2.

**Table 2:** Ablation results comparing MLP fusion with simple concatenation. Metrics are reported on the test set as mean ± standard deviation across five seeds. Best values per metric are highlighted in bold.

Setting	Accuracy	Precision	Recall	F1 Score	AUC
Fusion	0.91±0.01	0.86±0.01	0.99±0.01	0.92±0.01	0.95±0.005
Concat	0.76±0.02	0.72±0.04	0.88±0.07	0.79±0.02	0.88±0.01

Without the fusion MLP (simple concatenation), the model underperformed, with test accuracy ∼0.76 and AUC ∼0.88 across seeds. Conversely, including the fusion MLP substantially improved performance, raising accuracy to ∼0.91 and AUC to ∼0.95. Therefore, the fusion model was retained in the final model.

#### Data augmentation.

Additional flips and rotations reduced validation AUC in multi-seed runs. Therefore, augmentations were disabled in the final model to prevent degradation of generalization. The results of the ablation comparing the image-only baseline with and without data augmentation, over five independent seeds, are shown in Table 3.

**Table 3:** Ablation results comparing the performance on the test set with and without including data augmentations during training. The metrics are reported as mean ± standard deviation across five independent seeds. The best value for each metric is highlighted in boldface.

Setting	Accuracy	Precision	Recall	F1 Score	AUC
No aug	0.91±0.01	0.86±0.01	0.99±0.01	0.92±0.01	0.95±0.005
All augs	0.90±0.02	0.84±0.03	0.98±0.01	0.90±0.01	0.95±0.003

#### Surface preprocessing.

We compared models trained on normal versus smoothed interface representations, both in the image-only setting without augmentation. Results are summarized in Table 4. Both settings performed comparably, with no consistent advantage from smoothing.

**Table 4:** Ablation results comparing normal versus smoothed interface representations (image-only, no augmentation). Metrics are reported as mean ± standard deviation across five independent seeds. The best value for each metric is highlighted in boldface.

Setting	Accuracy	Precision	Recall	F1 Score	AUC
Normal	0.91±0.01	0.86±0.01	0.99±0.01	0.92±0.01	0.95±0.005
Smoothed	0.86±0.03	0.79±0.03	0.99±0.03	0.88±0.03	0.95±0.01

Overall, smoothing did not yield a consistent improvement over normal surfaces, so the final model retained the normal interface representation.

#### Input modality and descriptor contribution.

We next investigated whether geometric surface descriptors provided complementary information to the shape-based depth map baseline. The effect of the addition of each descriptor was evaluated individually over five independent seeds (Table 5). Metrics are reported on the test set (mean ± std across five seeds). Validation AUC is also included, as it served as the selection criterion in the greedy procedure.

**Table 5:** Ablation results comparing input modalities on the test set. Each model was trained without data augmentation using normal (non-smoothed) surfaces. Results are reported as mean ± standard deviation over five independent seeds. Best values per metric are highlighted in bold.

Input	Accuracy	F1 Score	Test AUC	Validation AUC
Image only	0.91±0.01	0.92±0.01	0.95±0.005	0.968±0.001
Img + *C*	0.87±0.03	0.89±0.02	0.96±0.005	0.977±0.002
Img + *F*_*den*2_	0.87±0.02	0.88±0.01	0.95±0.004	0.978±0.002
Img + *H*	0.87±0.03	0.89±0.02	0.96±0.003	0.977±0.003
Img + *K*	0.90±0.03	0.90±0.02	0.95±0.01	0.978±0.002
Img + *k*_1_	0.88±0.03	0.89±0.02	0.96±0.01	0.978±0.002
Img + *k*_2_	0.88±0.01	0.89±0.01	0.96±0.01	0.979±0.001
Img + *S*	0.89±0.02	0.90±0.02	0.94±0.01	0.970±0.003
Img + *sing*	0.89±0.01	0.90±0.01	0.97±0.005	0.978±0.003
Img + all	0.86±0.03	0.88±0.02	0.96±0.01	0.960±0.009

While the image-only model achieved strong baseline performance (AUC 0.95±0.005), several descriptors further improved the predictive accuracy. In particular, curvedness index (*C*), mean curvature (*H*) principal curvatures (*k*_1/2_), and *sing* consistently enhanced the AUC, whereas other descriptors such as *F*_*den*2_ and *S* had less pronounced or inconsistent effects. Notably, combining all descriptors jointly did not yield a substantial gain over individual descriptors and, in some cases, slightly reduced performance, suggesting that redundant or noisy channels may offset the benefits of informative ones.

To further isolate the most informative subset of descriptors, we performed a greedy forward feature selection procedure, starting from the image-only baseline and sequentially adding the descriptor that maximized validation AUC at each step. The first addition, principal curvature (*k*_2_), improved mean validation AUC from 0.968 to 0.979. At the default selection threshold (Δ=0.003), the procedure terminated after this step, identifying the image+*k*_2_ configuration as the optimal one. When the threshold was relaxed to Δ=0.0005, principal curvature *k*_1_ was added at step 2, yielding a marginal further increase to 0.980. Given the minimal improvement beyond *k*_2_, we retained image+*k*_2_ as the final descriptor configuration for subsequent experiments.

### Baseline comparison

Table 6 reports the results of our image-only CNN with sequence-based baselines.

**Table 6:** Comparison with sequence-only baselines. Metrics are calculated on the test set, as mean ± standard deviation over five seeds. Best values per metric are highlighted in boldface.

Setting	Accuracy	Precision	Recall	F1 Score	AUC
Image CNN	0.91±0.01	0.86±0.01	0.99±0.01	0.92±0.01	0.95±0.005
CDRs	0.69±0.02	0.79±0.08	0.53±0.09	0.62±0.05	0.76±0.02
H3	0.58±0.01	0.59±0.01	0.49±0.11	0.53±0.05	0.62±0.01
Fab	0.61±0.01	0.59±0.02	0.73±0.05	0.65±0.01	0.65±0.03

The best sequence-based Siamese baseline (all CDRs with ESM-3 embeddings) achieved AUC 0.765±0.021 (accuracy 0.686±0.021). Whole-chain and H3-only variants performed worse, with AUC values of 0.653±0.031 and 0.625±0.007, respectively. In contrast, our structure-based Siamese CNN consistently achieved an AUC of at least ~0.95. These results highlight the added value of surface geometry: explicit 3D surface representations provide substantially stronger discrimination of binding versus non-binding interfaces.

### Final model performance

**Fig 4:**
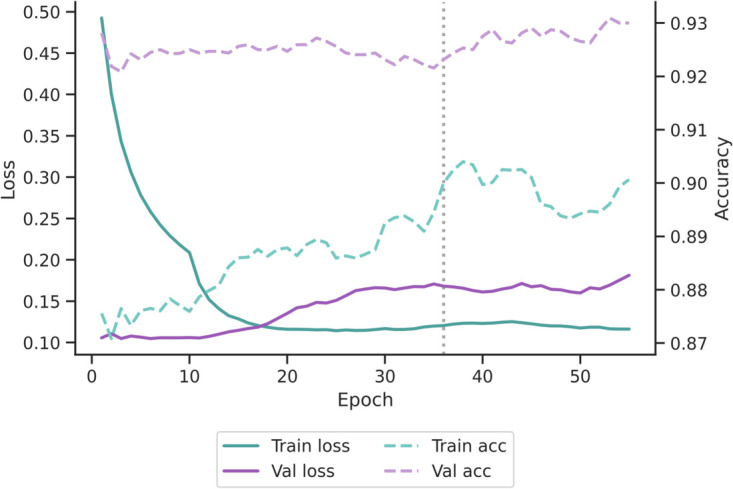
Training and validation curves for the final selected model (image + *k*_2_ setting, best validation seed). Solid lines show training and validation losses (left axis), while dashed lines show accuracies (right axis). As expected, training loss remains lower than validation loss. Validation accuracy is slightly higher than training accuracy across epochs; this arises because accuracy is computed after applying an adaptive ROC-based threshold, whereas loss reflects continuous margins between positive and negative pairs. Training was extended to 100 epochs with early stopping (patience = 20), ensuring convergence and preventing overfitting. The early stopping epoch (36) is indicated with a grey dashed vertical line.

During training, we monitored loss and accuracy metrics for the final selected configuration (image + *k*_2_). Fig 4 shows the trends of training and validation loss (solid lines) and accuracy (dashed lines), using the seed that achieved the highest validation ROC AUC. Loss curves display the expected behavior, with training loss lower than validation loss. As accuracy is threshold-dependent, while loss reflects the continuous margin between positive and negative pairs, the adaptive ROC-based threshold used during validation favored higher classification accuracy, despite a slightly larger validation loss, resulting in consistently high validation accuracy.

**Fig 5:**
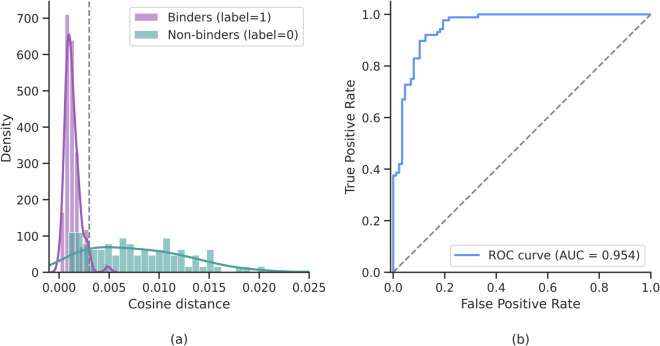
Test-set separation of binding and non-binding pairs. (a) Distribution of cosine distances between embeddings of binding (purple) and non-binding (teal) pairs in the test set. Binder pairs show lower distances, consistent with geometric complementarity. The vertical dashed line indicates the decision threshold optimized on the validation set. (b) Receiver Operating Characteristic (ROC) curve for the same test set, with area under the curve (AUC) reported. The curve demonstrates strong discrimination between classes, with an AUC over 0.95.

To further assess how the model distinguishes between binding and non-binding pairs, we analyzed the distribution of distances between embeddings on the test set and compared them with the corresponding Receiver Operating Characteristic (ROC) curve. This analysis, shown in Fig 5, complements the training and validation trends by showing the degree of separation achieved in the learned representation space and the resulting classification performance across thresholds.

Taken together, these results demonstrate that the proposed Siamese CNN consistently outperforms sequence-based baselines and benefits from the selected architectural and input design choices. The ablation experiments confirmed that cosine loss and the fusion module were beneficial for robust performance, while smoothing and heavy augmentation were not. Among descriptors, *k*_2_ emerged as the most informative complement to depth maps, providing a simple yet effective final configuration. The final model achieved clear separation of embedding distances and good ROC performance on the held-out test set. These findings underscore the effectiveness of geometry-aware representations.

## Conclusion

This work addressed the challenge of classifying RBD–antibody interfaces as binding or non-binding pairs using geometric and image-based deep learning. We proposed a Siamese convolutional neural network trained on depth-map renderings of protein interfaces, optionally combined with geometric surface descriptors. The model was optimized with a cosine contrastive loss and evaluated on homology-aware splits to prevent information leakage across folds. Our ablation experiments clarified the design choices underlying the final model. Data augmentation with flips and rotations reduced generalization, and surface smoothing was not beneficial. In contrast, the fusion module significantly improved cross-modal integration, and cosine contrastive loss aligned well with normalized embeddings. Among individual descriptors, the mean principal curvature *k*_2_ emerged as the most effective, representing the strongest improvement over the baseline. Curvature information is biologically intuitive in this context, as *k*_2_ encodes local convexity and concavity patterns that are relevant for complementarity. Other descriptors provided moderate improvements but fell short of *k*_2_. For example, the Shape Index *S* captures qualitative surface geometry (peaks, ridges, saddles, valleys) that is known to influence binding affinity, yet it performed slightly worse than *k*_2_ in our benchmarks. Similarly, *K* and *F*_*den*2_ emphasize global surface characteristics or isolated critical points, which may be less discriminative for antibody binding sites. Principal curvature *k*_1_ was also evaluated and, while informative, added only marginal benefit beyond *k*_2_ under a greedy selection procedure. These findings underscore the importance of choosing descriptors aligned with the biological context of protein recognition. Local curvature features complement the global depth-map representation and provide a meaningful signal. This suggests that future work could extend the framework by incorporating descriptors of physicochemical properties (e.g., electrostatics or hydrophobicity) alongside geometric cues, thereby improving generalizability and robustness. Compared to a sequence-only baseline using ESM-3 embeddings, our structure-based approach achieved substantially higher performance (ROC–AUC ∼0.95 vs ∼0.76), underscoring the added value of 3D geometric information. Our results highlight the potential of geometric deep learning for practical antibody discovery. Accurate discrimination between binding and non-binding antigen–antibody interfaces can accelerate therapeutic antibody development by prioritizing promising Fab candidates, and can inform vaccine design by identifying stable immunogenic epitopes. These findings serve as a proof-of-concept for the translational potential of integrating artificial intelligence with structural bioinformatics, paving the way for broader applications in biomedical and drug discovery research. While broader applications are possible, this study’s primary focus was to improve predictive performance within COVID-19-related antibody interactions. The good test performance confirms that the model effectively generalizes within this focused dataset, aligning with the intentional design choice to target this specific biological challenge. Additionally, the method relies on experimentally solved antigen-antibody complexes, where binding interfaces are well-defined. While this ensures precise characterization of binding site geometry, it does not explicitly account for protein flexibility or binding-induced conformational changes. Future work could explore integrating docking simulations or flexible protein modeling to extend this method to unbound structures. Incorporating these strategies could improve the model’s applicability to cases where experimental structures are unavailable or incomplete. Finally, increasing data complexity by incorporating additional types of information across multiple channels and using higher-resolution images may refine the model’s ability to distinguish between complex and non-complex pairs.
